# Gut Microbiota Composition and Structure of the Ob/Ob and Db/Db Mice

**DOI:** 10.1155/2019/1394097

**Published:** 2019-03-11

**Authors:** Mingsheng Yang, Yixin Liu, Hengchang Xie, Zhengzheng Wen, Yunxia Zhang, Changjing Wu, Li Huang, Jie Wu, Chensheng Xie, Tao Wang, Weifeng Peng, Shangqi Liu, Long Chen, Xiaomeng Liu

**Affiliations:** ^1^Institute of Neuroscience and Translational Medicine, College of Life Science and Agronomy, Zhoukou Normal University, Zhoukou, Henan 466001, China; ^2^College of Science, Hangzhou Normal University, Hangzhou, Zhejiang 311121, China

## Abstract

**Introduction:**

Gut microbiota is involved in the progression of metabolic diseases such as obesity and type 2 diabetes. The ob/ob and db/db mice are extensively used as models in studies on the pathogenesis of these diseases. The goal of this study is to characterize the composition and structure of gut microbiota in these model mice at different ages.

**Materials and methods:**

High-throughput sequencing was used to obtain the sequences of the highly variable 16S rRNA V3-V4 region from fecal samples. The taxa with high abundance in both model mice were identified by bioinformatics analysis. Moreover, the taxa with divergent abundance in one model mice at different ages or in both model mice at the same age were also recognized.

**Discussion and conclusion:**

The high abundance of Bacteroidetes and Firmicutes in microbiota composition and their imbalanced ratio in both model mice reflect the state of metabolic disorders of these mice. Differences in microbiota composition between the two model mice of the same age or in one model mice with different ages were assumed to be closely linked to the fluctuation of their blood glucose levels with age. The data on gut microbiota in ob/ob and db/db mice investigated herein has broad implications for the pathogenesis study and drug discovery on obesity and related complications.

## 1. Introduction

Gut microbiota represents a microbial community resident in the human intestine that exerts a crucial impact on human health. The prevalence of obesity and type 2 diabetes (T2D) worldwide is increasing, which has brought huge medical costs to society in past decades [1–3]. Thus, studies on the link between gut microbiota and obesity and T2D have been intensely conducted in recent years. Epidemiological studies demonstrated that the microbial composition of individuals with T2D is quite different from their normal controls, and it is characterized by a lower abundance of universal butyrate-producing bacteria and an increase in opportunistic pathogens [4, 5]. In model animals, Backhed et al. [6] reported that germ-free mice which received microbiota from normal mice produced a 60% increase in body fat content 14 days later, even though they consumed less food than their germ-free controls. In diet-induced obesity, low-grade inflammation was regarded as a factor contributing to obesity-related metabolic disorders such as T2D [7]. Further studies clarified that a high-fat diet can change microbiota composition and increase intestinal permeability. Consequently, more lipopolysaccharides (LPS) are released from the gut, which initiates toll-like receptor-4 (TLR4) signaling and inflammatory response, contributing to the development of obesity and T2D [8–10]. Collectively, the aforementioned evidence indicates that gut microbiota is closely associated with the progression of obesity and T2D.

Leptin is mainly produced by adipocytes, and it plays a crucial role in the regulation of energy homeostasis, skeletal growth, and the immune responses [11–13]. Impaired leptin signaling is closely associated with metabolic diseases, such as obesity and type 2 diabetes. Ob/ob mice are genetically obese animals because their leptin-encoding gene has a systemic nonsense and is biologically inactive [14]. In contrast, in db/db mice, the receptor-encoding sequence of leptin is mutated, rendering these mice with high circulatory levels of leptin but lacking intracellular leptin action [15]. Hence, db/db mice are characterized as genetically diabetic. Interestingly, previous studies have recognized that the blood glucose levels of ob/ob and db/db mice fluctuate with age. In ob/ob mice, hyperglycemia can be observed from 4 weeks of age, but this state starts to decline after 12 weeks and blood glucose levels are comparable to control mice until 28 weeks [16, 17]. In contrast, db/db mice develop hyperglycemia at 8 weeks of age, and this state is not transient later [17, 18].

Given the prevalence of obesity and T2D worldwide, great efforts are being made in clarifying its pathogenesis and in screening the drugs of these diseases. In these efforts, ob/ob and db/db mice are routinely used as model organisms (e.g., Wang et al. [19], Liu et al. [20], and Ahmad et al. [21]). The close link between gut microbiota and these metabolic diseases indicates that understanding the gut microbiota composition and structure of ob/ob and db/db mice is very indispensable. Geurts et al. [22] reported the difference of the gut microbiota between six-week-old db/db and lean mice, emphasizing the specific relationships between the gut microbiota and the regulation of the apelinergic system. Most other studies on this issue only sequenced and analyzed the gut microbiota of the model mice at a specific age [23–25]. However, the dynamic changes of gut microbiota with age of model mice have seldom been investigated. In this study, we set out to investigate the composition and structure of gut microbiota in ob/ob and db/db mice as well as their dynamic change with age using a high-throughput sequencing method. Our results would provide broad implications for further investigations on the pathophysiology and pharmacology of obesity and T2D.

## 2. Materials and Methods

### 2.1. Animals

Six-week-old male ob/ob (*n* = 3) and db/db (*n* = 3) mice in a C57BL/6J background were purchased from The Jackson Laboratory (Bar Harbor, ME). Animals were maintained in an atmosphere controlled at 22 ± 2°C and 55 ± 10% relative humidity with a 12 h light and 12 h dark cycle. Animals were housed under barrier-maintained conditions with free access to water and a normal chow diet. Body weight was measured biweekly. All animal experiments were approved by the Animal Ethics Committee (approval ID: ZKNU2017023) of Zhoukou Normal University.

### 2.2. Fecal Collection and DNA Extraction

Total metagenomic DNA was extracted from fecal samples using the QIAmp Fast DNA Stool Mini Kit according to the manufacturer's protocol (cat. no. 51604; Qiagen, Hilden, Germany). Fecal samples were collected from 3 mice per group, and each group included the ob/ob or db/db mice at 8, 12, or 18 weeks of age. DNA quantification was performed on a NanoDrop 2000 Spectrophotometer (Thermo Fisher Scientific Inc.).

### 2.3. High-Throughput Sequencing of 16S rRNA Genes and Analysis

High-throughput sequencing was performed with the Illumina HiSeq PE250 platform at the Shanghai Ruiyi Biotechnology Co. Ltd. (Shanghai, China). Amplification of the highly variable V3-V4 region of the 16S rRNA gene was conducted using universal primers 341F (5′-CCTACGGGRSGCAGCAG-3′) and 806R (5′-GGACTACVSGGGTATCTAAT-3′). Raw paired-end reads were assembled by PANDAseq, and the following conditions were used to define the quality filter: reads with an average quality score of more than 20, reads with less than three ambiguous N bases, and reads with a long range from 220 to 500 nucleotides [26]. After the singletons were discarded, the sequences were clustered into operational taxonomic units (OTUs) using the software USEARCH at ≥97% similarity [27]. Linear discriminant analysis (LDA) effect size (LEfSe) was conducted to assess the effect size of each differentially abundant OUT [28]. Subsequent bioinformatics steps were analyzed using a QIIME software package [29].

### 2.4. Statistical Analysis

Statistical analysis on the body weight of animals was performed using GraphPad Prism version 6 (San Diego, CA). All data are expressed as means ± SEM. In LEfSe analysis, the parameters of wilcox.test and kruskal.test were used to define the differences in the relative abundance of bacteria between groups.

## 3. Results

### 3.1. Body Weight of Ob/Ob and Db/Db Mice

**Figure 1:**
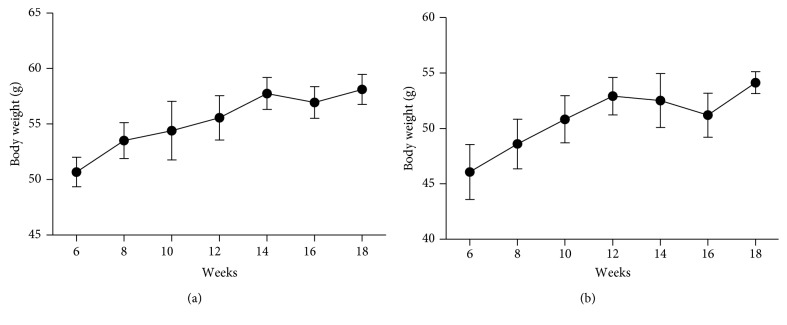
Body weight of the ob/ob mice (a) and db/db mice (b).

To examine the phenotype change with age of the ob/ob and db/db mice, we measured the body weight of the model mice aged from 6 to 18 weeks. The average body weight of the 4 ob/ob mice measured consistently increased with age except for that of mice at 16 weeks of age (Figure 1(a)). There was almost an 8 g difference in body weight between the 6-week and 18-week age groups, with the former weighing 50.68 ± 1.33 g and the latter 58.12 ± 1.35 g. A tendency for the increased average body weight of the 4 db/db mice can be observed during the treated time, although there is a slight decrease at 14 and 16 weeks relative to the mice aged 12 weeks (Figure 1(b)). At the end of the experiment, the average body weight was 58.12 ± 1.00 g, an increase of about 8 g relative to the initial 46.07 ± 2.46 g.

### 3.2. Fecal Microbiota Composition

**Figure 2:**
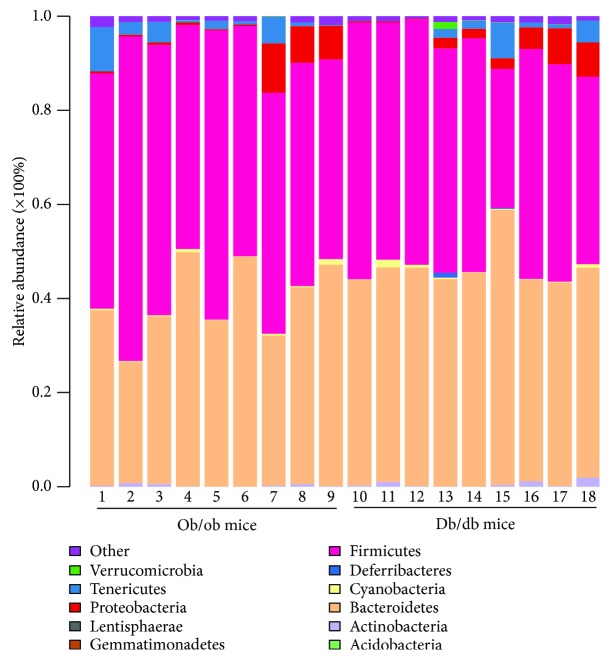
Relative abundance of the major OTUs at the phyla level of the fecal samples from ob/ob and db/db mice.

**Figure 3:**
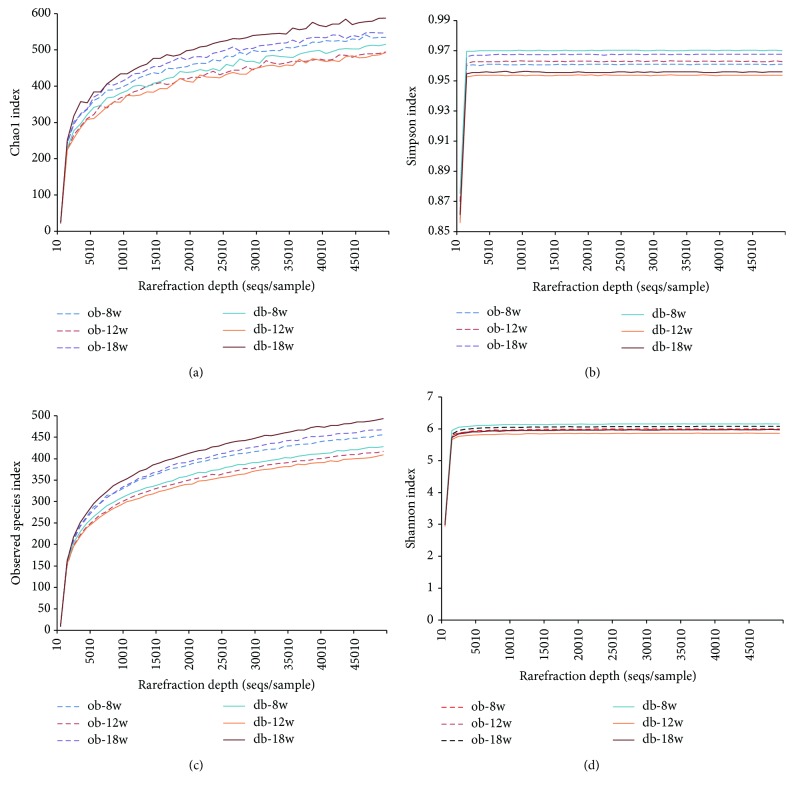
*α*-Diversity of fecal microbiota in different groups as shown by Chao1 index (a), Simpson index (b), observed species index (c), and Shannon index (d).

Based on the Illumina MiSeq sequencing platform, the highly variable V3-V4 regions of the 16S rRNA gene from 18 samples were sequenced. A clustering analysis based on a 97% similarity identified 14,292 operational taxonomic units (OTUs). As shown in Figure 2 and Tables 1 and 2, of the 11 microbial phyla defined by these OTUs, Bacteroidetes and Firmicutes dominate the microbial composition in ob/ob mice, and show 39.28% and 52.86% relative abundance on average, respectively. In db/db mice, they also exhibit relatively high abundance, with an average of 46.06% and 46.58%, respectively. This was followed by Proteobacteria, Tenericutes, Actinobacteria, and Cyanobacteria, with a relative abundance of 3.08%, 2.9%, 0.3%, and 0.38% in ob/ob mice, respectively, and 2.96%, 1.75%, 0.57%, and 0.44% in db/db mice, respectively. The other five phyla, including Acidobacteria, Deferribacteres, Gemmatimonadetes, Lentisphaerae, and Verrucomicrobia, showed extremely low abundance in both ob/ob and db/db mice. Overall, no significant difference can be recognized in the *α*-diversity of fecal microbiota from one model animal with different ages or the two model animals of the same age (Figure 3).

### 3.3. The Change of Microbial Compositions in Ob/Ob and Db/Db Mice with Age

**Figure 4:**
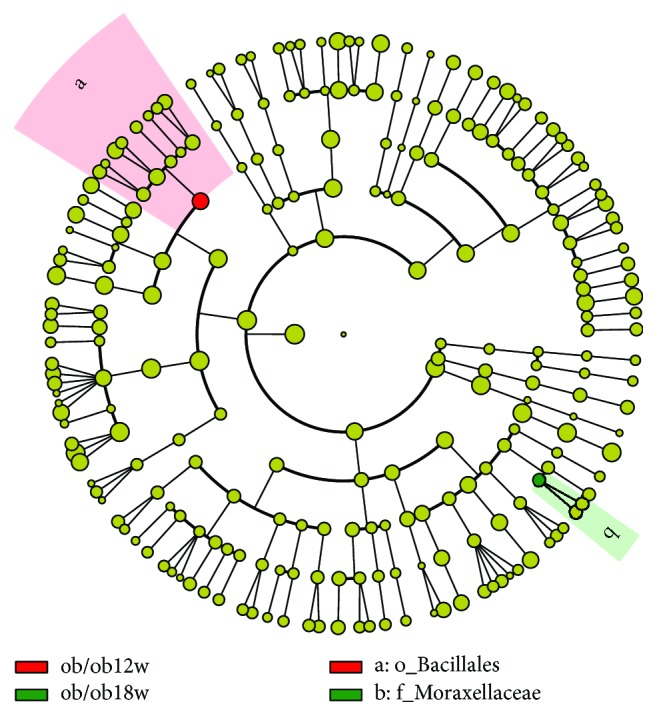
Cladogram representing a significantly high abundance of taxa in the gut microbiota of ob/ob mice at 12 and 18 weeks of age.

**Figure 5:**
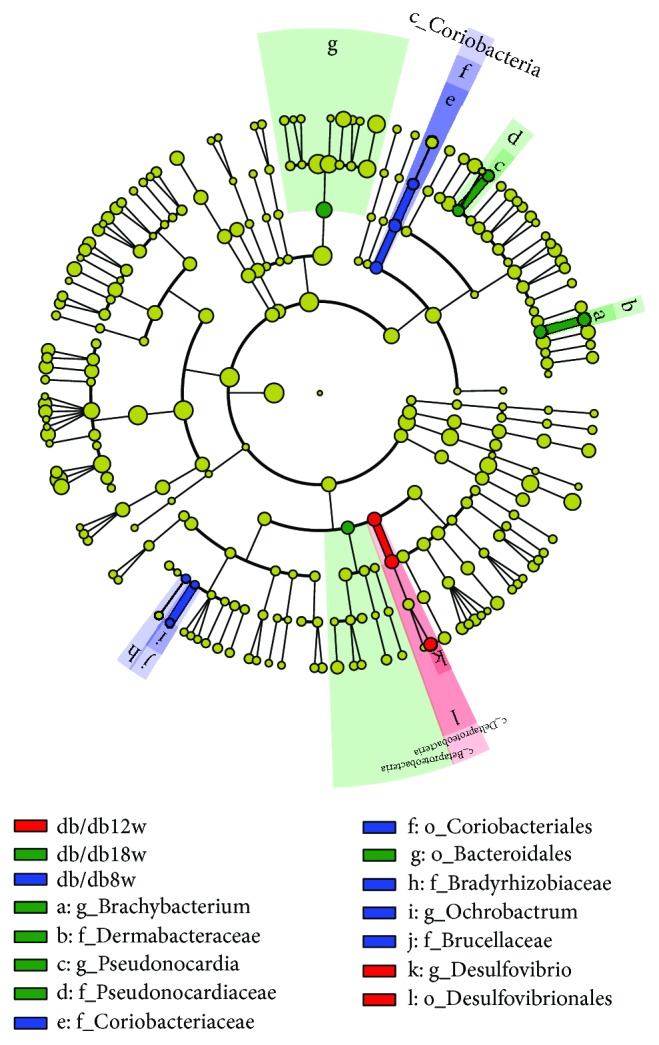
Cladogram representing a significantly high abundance of taxa in the gut microbiota of db/db mice at 8, 12, and 18 weeks of age.

Gut microbial compositions in both ob/ob and db/db mice changed with increasing age. With the LDA score > 2 as the threshold for significance, LefSe analysis revealed that the *Bacillales* of fecal microbiota from 12-week-old ob/ob mice was significantly high relative to that of another age group. However, the abundance of Moraxellaceae in mice aged 18 weeks was significantly higher than that of younger ob/ob mice investigated in this study (Figure 4). In db/db mice, the Coriobacteriaceae, *Ochrobactrum*, and Bradyrhizobiaceae of fecal microbiota from 8 weeks of age were significantly high relative to that of other two age groups. In 12-week-old db/db mice, a significant enrichment to the taxa was *Desulfovibrio*. However, the abundance of *Brachybacterium*, *Pseudonocardia*, and Bacteroidales in mice aged 18 weeks was significantly higher than that of younger db/db mice investigated in this study (Figure 5).

### 3.4. Comparison of Microbial Compositions between the Ob/Ob and Db/Db Mice of the Same Age

**Figure 6:**
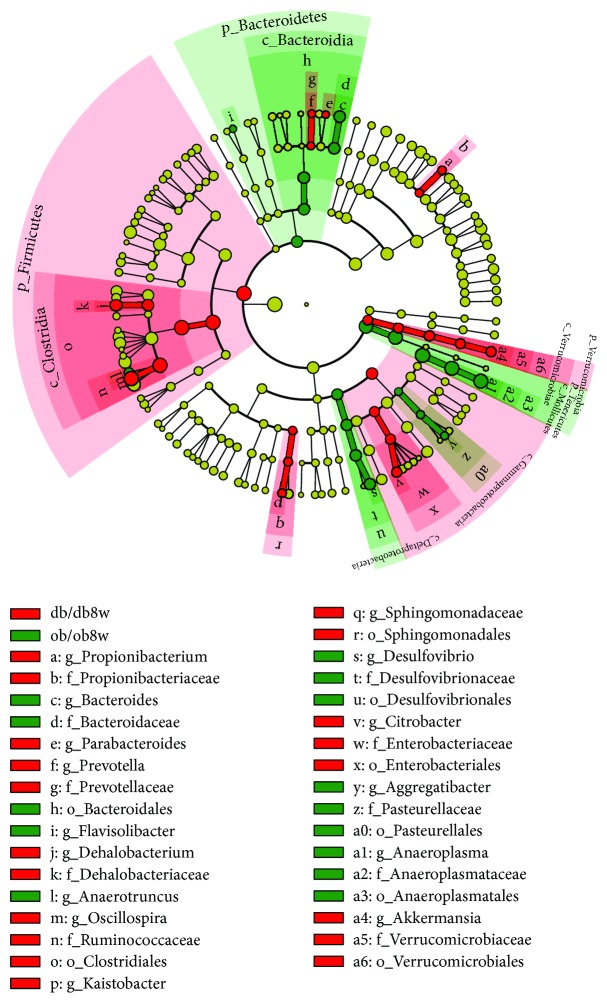
Cladogram representing a significantly high abundance of taxa in the gut microbiota of ob/ob and db/db mice at 8 weeks of age.

**Figure 7:**
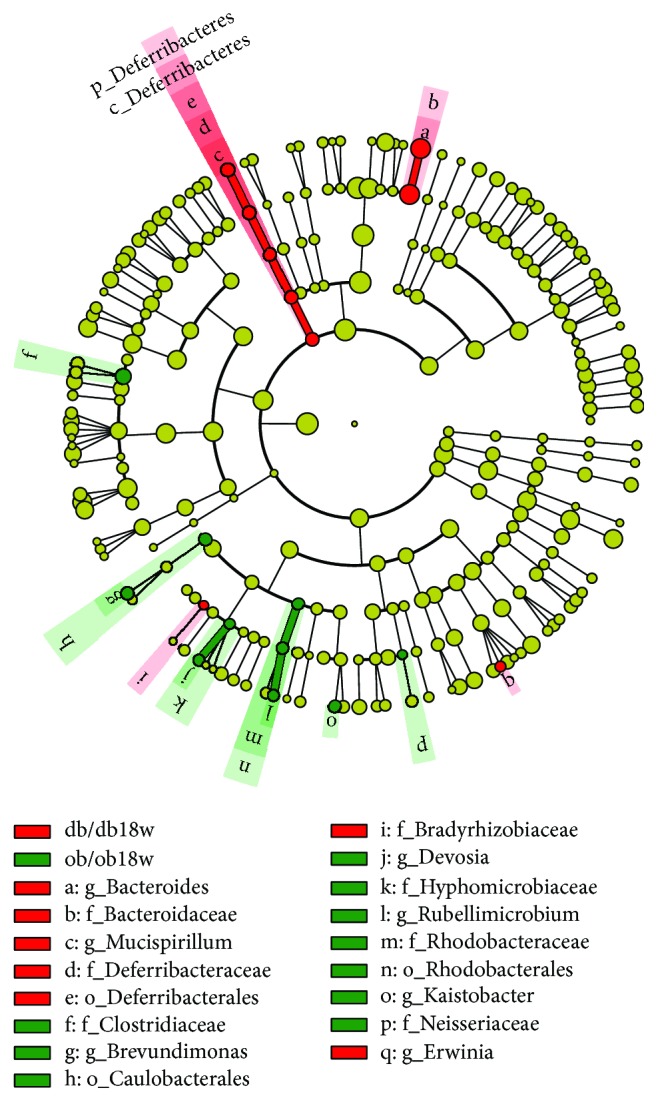
Cladogram representing a significantly high abundance of taxa in the gut microbiota of ob/ob and db/db mice at 18 weeks of age.

With the LDA score > 2 as the threshold for significance, LefSe analysis revealed that there were significant differences in microbial composition between ob/ob and db/db mice at the same age. At 8 weeks of age, a significant enrichment to the taxa in ob/ob mice were Bacteroidaceae, Desulfovibrionaceae, Pasteurellaceae, and Anaeroplasmataceae at the family level and *Bacteroides*, *Flavisolibacter*, *Anaerotruncus*, *Desulfovibrio*, *Aggregatibacter*, and *Anaeroplasma* at the genus level. In db/db mice with the same age, a significant enrichment to the taxa were Propionibacteriaceae, Prevotellaceae, Dehalobacteriaceae, Ruminococcaceae, Sphingomonadaceae, Eterobacteriaceae, and Verrucomicrobiaceae at the family level and *Propionibacterium*, *Parabacteroides*, *Prevotella*, *Dehalobacterium*, *Oscillospira*, *Kalstobacter*, *Citrobacter*, and *Akkermansia* at the genus level (Figure 6). As shown in Figure 7, when the mice were 18 weeks of age, larger proportions of *Brevundimonas*, *Devosia*, *Rubellimicrobium*, and *Kaistobacter* at the genus level were found in ob/ob mice, whereas *Bacteroides*, *Mucispirillum*, and *Erwinia* at the genus level were the significant enrichment to the taxa in db/db mice.

## 4. Discussion

The ob/ob and db/db mice are extensively employed as animal models in studies on the pharmacology and pathogenesis of metabolic diseases such as obesity and type 2 diabetes [17]. Given the involvement of gut microbiota in the pathogenesis of these diseases, we provide the structure and composition of gut microbiota in ob/ob and db/db mice at different ages in the present study. To our knowledge, this is the first study focusing on the gut microbiota of the ob/ob and db/db mice at different ages, and these data would provide broad implications for the management of obesity and related complications.

Our study shows that the abundance of Bacteroidetes and Firmicutes is the highest in microbiota composition from both ob/ob and db/db mice, reaching 42.67% and 49.72% on average, respectively. These values are comparable to that of either the ob/ob and db/db mice or the normal C57Bl/6J mice provided in other studies [30, 31]. Moreover, a series of studies have observed that the ratio of Firmicutes/Bacteroidetes is positively correlated with obesity and glucose tolerance severity [30, 32, 33]. In this study, the ratios of Bacteroidetes/Firmicutes are 1.35 in ob/ob mice and 1.01 in db/db mice, which are evidently higher than that of normal C57Bl/6J mice investigated in other studies (i.e., Roopchand et al., [34]), indicating that the model mice we used are indeed under the state of metabolic disorders. As expected, the microbial compositions between the ob/ob and db/db mice with the same age are quite different, which may be associated with their different genetic background. Indeed, ob/ob and db/db mice are also used as different animal models in studies (e.g., Park et al. [35] and Jia et al. [36]).

Gut microbiota play a crucial role in glucose homeostasis and some beneficial bacteria have been identified such as the genera *Akkermansia* [34, 37–39] and *Oscillospira* [38]. These recognized taxa have promoted the development of gut microbiota-based agents against insulin resistance and other metabolic disorders. In the present study, the fluctuation of the blood glucose levels of ob/ob and db/db mice with age reported before prompted us to suppose that the gut microbiota composition in them may be altered with age as well. Interestingly, our study revealed that microbial composition showed a similar fluctuation pattern with that of the blood glucose values in ob/ob and db/db mice with age. This reflects that the gut microbiota, especially the taxa with a relatively higher abundance at a specific age, may be involved in glucose homeostasis. However, additional work is needed to establish whether and how these taxa functionally modulate the glucose homeostasis, providing microbiota recourse for developing novel therapies against obesity and associated metabolic disorders.

## 5. Conclusions

The ob/ob and db/db mice are extensively employed as models in studies on the pathogenesis and drug discovery of metabolic diseases such as obesity and type 2 diabetes. In the present study, we sequenced and analyzed the gut microbiota of the model mice across different ages. Our results showed that the microbial composition exhibits a similar fluctuation pattern with that of the blood glucose values in ob/ob and db/db mice with age. The microbial taxa with a relatively higher abundance at the specific age identified herein would provide microbiota recourse for the development of novel therapies against obesity and associated metabolic disorders.

## Figures and Tables

**Table 1:** Relative abundance in the percentage of the major OTUs at the phyla level in each fecal sample from ob/ob mice.

Taxa	Ob/ob 8 weeks			Ob/ob 12 weeks			Ob/ob 18 weeks		
	1	2	3	4	5	6	7	8	9
Acidobacteria	0.000	0.000	0.000	0.000	0.000	0.000	0.004	0.000	0.000
Actinobacteria	0.242	0.771	0.579	0.064	0.034	0.057	0.256	0.564	0.129
Bacteroidetes	37.284	25.747	35.628	49.829	35.351	48.849	31.891	41.835	47.063
Cyanobacteria	0.316	0.221	0.247	0.658	0.140	0.083	0.366	0.267	1.194
Deferribacteres	0.000	0.000	0.000	0.000	0.000	0.000	0.000	0.000	0.000
Firmicutes	50.013	68.955	57.492	47.609	61.484	48.953	51.254	47.489	42.448
Gemmatimonadetes	0.000	0.000	0.000	0.001	0.000	0.000	0.005	0.000	0.000
Lentisphaerae	0.000	0.000	0.000	0.000	0.000	0.000	0.000	0.000	0.000
Proteobacteria	0.412	0.381	0.488	0.520	0.247	0.281	10.473	7.739	7.142
Tenericutes	9.463	2.679	4.369	0.472	1.776	0.714	5.650	0.791	0.157
Verrucomicrobia	0.000	0.000	0.000	0.062	0.000	0.000	0.077	0.000	0.000
Others	2.269	1.247	1.196	0.785	0.967	1.062	0.024	1.314	1.867

Note: “ob/ob 8 weeks” indicates the group of ob/ob mice at 8 weeks of age; “ob/ob 12 weeks” indicates the group of ob/ob mice at 12 weeks of age; “ob/ob 18 weeks” indicates the group of ob/ob mice at 18 weeks of age.

**Table 2:** Relative abundance in the percentage of the major OTUs at the phyla level in the fecal sample from db/db mice.

Taxa	Db/db 8 weeks			Db/db 12 weeks			Db/db 18 weeks		
	1	2	3	4	5	6	7	8	9
Acidobacteria	0.000	0.000	0.000	0.000	0.000	0.000	0.000	0.000	0.001
Actinobacteria	0.288	1.038	0.126	0.045	0.029	0.384	1.155	0.123	1.920
Bacteroidetes	43.714	45.579	46.454	44.068	45.419	58.519	42.867	43.287	44.648
Cyanobacteria	0.085	1.656	0.583	0.337	0.145	0.163	0.152	0.172	0.699
Deferribacteres	0.000	0.000	0.000	1.032	0.002	0.226	0.002	0.002	0.047
Firmicutes	54.579	50.412	52.253	47.711	49.731	29.526	48.879	46.243	39.874
Gemmatimonadetes	0.000	0.000	0.000	0.000	0.001	0.000	0.000	0.000	0.000
Lentisphaerae	0.000	0.000	0.000	0.000	0.000	0.000	0.000	0.000	0.000
Proteobacteria	0.287	0.197	0.155	2.243	2.025	2.271	4.585	7.637	7.231
Tenericutes	0.007	0.003	0.003	1.852	1.696	7.547	1.039	0.754	4.651
Verrucomicrobia	0.016	0.031	0.001	1.498	0.091	0.061	0.002	0.091	0.004
Others	1.023	1.083	0.424	1.213	0.861	1.304	1.318	1.691	0.925

Note: “db/db 8 weeks” indicates the group of db/db mice at 8 weeks of age; “db/db 12 weeks” indicates the group of db/db mice at 12 weeks of age; “db/db 18 weeks” indicates the group of db/db mice at 18 weeks of age.

## Data Availability

The data used to support the findings of this study are available from the corresponding author upon request.
